# Binding Energy and Diffusion Barrier of Formic Acid on Pd(111)

**DOI:** 10.1021/acs.jpca.2c07414

**Published:** 2022-12-30

**Authors:** Jan Fingerhut, Loïc Lecroart, Dmitriy Borodin, Michael Schwarzer, Stefan Hörandl, Alexander Kandratsenka, Daniel J. Auerbach, Alec M. Wodtke, Theofanis N. Kitsopoulos

**Affiliations:** † Institute for Physical Chemistry, Georg-August University of Goettingen, Goettingen 37077, Germany; ‡ Department of Dynamics at Surfaces, Max Planck Institute for Multidisciplinary Sciences, Goettingen 37077, Germany; § International Center for Advanced Studies of Energy Conversion, Georg-August University of Goettingen, Goettingen 37077, Germany; ∥ Department of Chemistry, University of Crete, Heraklion 715 00, Greece; ⊥ Institute of Electronic Structure and Laser − FORTH, Heraklion 70013, Greece

## Abstract

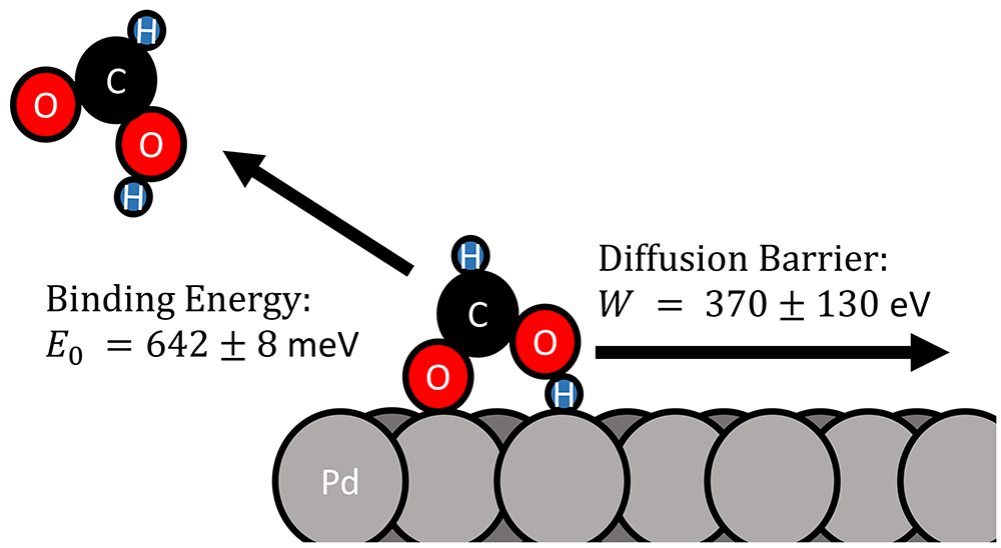

Velocity-resolved kinetics is used to measure the thermal rate of formic acid desorption from Pd(111) between 228 and 273 K for four isotopologues: HCOOH, HCOOD, DCOOH, DCOOD. Upon molecular adsorption, formic acid undergoes decomposition to CO_2_ and H_2_ and thermal desorption. To disentangle the contributions of individual processes, we implement a mass-balance-based calibration procedure from which the branching ratio between desorption and decomposition for formic acid is determined. From experimentally derived elementary desorption rate constants, we obtain the binding energy 639 ± 8 meV and the diffusion barrier 370 ± 130 meV using the detailed balance rate model (DBRM). The DBRM explains the observed kinetic isotope effects.

## 1 Introduction

The knowledge of binding energies of molecules at catalytic metal surfaces has enormous importance in the evaluation of catalytic activity and as benchmarks for quantum chemical calculations. However, up to now only a few molecule/metal surface binding energies are known from accurate enough experiments to allow meaningful comparison to state-of-the-art electronic structure calculations. Recently 39 molecule/metal systems were summarized that are believed to serve as good benchmarks.^1^ Despite more recent extensions of this data set,^2^ many of the broad range electronic structure studies^3,4^ have focused their effort predominantly to comparison of these systems reported by Wellendorff et al.^1^ Close inspection of these systems reveals a lack of chemical diversity. This table contains nine CO binding energies, five dissociative adsorption energies for H_2_, and four entries for NO, O_2_, and benzene. We are in danger of letting these five molecules dominate our thinking about how different exchange correlation (XC) functionals perform for the predictions of binding energies in heterogeneous catalysis. Molecules like N_2_, NH_3_, and HCOOH, despite their paramount importance for industrial heterogeneous catalysis, are rare guests of such benchmark tables, which leads to a missing intuition of their magnitude.

Of course, molecular binding at surfaces is associated with different bond types, and correlating the performance of XC functionals to the magnitude of the binding energy appears courageous at best. For example, strong bonds between transition metals and CO have contributions from sigma-bonding as well as back-bonding, where electron density from the metal is pushed into the 2π* orbital of the molecule. This covalent bond remains a challenge for GGA-DFT functionals, which must accurately describe both of these two types of bonding.^5^ Another example is the adsorption of benzene at metal surfaces, which is strongly influenced by van der Waals interactions, and for some metals an XC functional like PBE and RPBE would not consider benzene bound at all.^1^

Although many methods have been applied to determine binding energies, single crystal adsorption calorimetry (SCAC) in combination with sensitive pyroelectric detectors has proven itself to be one of the most reliable tools.^6−8^ A special challenge arises for binding energies of highly reactive molecule/surface systems. For SCAC, experimental conditions have to be carefully chosen such that either molecular adsorption or decomposition reactions dominate; only then can the heat release be used to derive binding energies. In fact, the quantification of binding energies under conditions where molecular desorption competes with decomposition has rarely been achieved.

Recently, the velocity-resolved kinetics (VRK) method^9^ has demonstrated determinations of adsorption energies that are at least as accurate as those obtained with SCAC.^10−12^ By measuring accurate desorption rates, statistical rate modeling and detailed balance can be applied to obtain binding energies. In this work, we demonstrate the capability of VRK to determine binding energies in reactive systems; specifically, we present work from which the binding energy of formic acid on Pd(111) is derived, under conditions where molecular desorption competes with decomposition to CO_2_ and H_2_. We directly measure the temperature-dependent formic acid surface lifetime, and through an independent determination of the formic acid desorption probability, we obtain the corresponding elementary rate constant for formic acid desorption. The derived desorption rate constant in combination with accurate adsorbate entropy modeling allows us to derive the binding energy and diffusion barrier of formic acid on Pd(111). We applied this approach to four isotopologues to obtain benchmarks for electronic structure theory.

## 2 Methods

### 2.1 Experimental Section

The apparatus has been described previously in detail.^13^ A molecular beam of formic acid is produced by bubbling helium through each of the formic acid isotopologues at room temperature and expanding this gas mixture (∼1% formic acid in He) through a pulsed nozzle operating at 25 Hz. Each molecular beam pulse doses the surface with (1.0 ± 0.3) × 10^–4^ monolayer (ML) of formic acid. The absence of impurities and a constant formic acid flux during the experiment is verified by a mass analysis of the molecular beam.^13^ The supersonic molecular beam pulse (25 μs FWHM) passes through two differentially pumped stages, enters the surface-scattering chamber with a base pressure of 2 × 10^–10^ mbar, and impinges upon the Pd surface (MaTeck GmbH) at an incidence angle of 30° to the surface normal. We use a Pd(111) single crystal whose step density is estimated from the miscut angle to be on the order of 0.1–0.2%. The Pd surface is prepared by sputtering with Ar^+^ (3 keV) for 15 min, subsequent annealing at 1023 K for 15 min followed by annealing at 1173 K for 3 min, and verification of surface cleanliness using Auger electron spectroscopy.

The propagation directions of the molecular beam and the ionizing probe laser define the scattering plane; the normal direction of the scattering plane is oriented parallel to the Pd crystal face. The normal direction of the scattering plane points to an ion imaging detector. Desorbing molecules are ionized 20 mm above the surface, using a Ti:sapphire laser (35 fs, 0.5 W at 1 kHz) via nonresonant multiphoton ionization (MPI). A pulsed homogeneous electric field pointing along the normal direction of the scattering plane, projects the nascent ions onto a microchannel plate (MCP)–phosphor anode imaging detector. The mass-to-charge ratio of the ions is selected by time-gating the MCP gain with respect to the pulsed-field extraction. Ion images appearing on the phosphor screen are recorded using a CCD camera.

The flux of desorption products as a function of time is measured by recording the product density as a function of the time delay between the ionizing laser and the pulsed molecular beam. Velocities of both the reactants and the scattered/desorbing products are obtained from ion images and are used to convert the observed product density to flux. The distance of laser from the surface and the measured velocities determine the flight time of the desorption products from the surface to the laser ionization region. This flight time is subtracted from the time delay between the ionizing laser and the pulsed molecular beam. The arrival time of the formic acid at the surface is determined in a separate experiment. This information is used to determine absolute residence times and desorption fluxes,^9^ the “kinetic trace”.

Kinetic traces for HCOOH, DCOOH, HCOOD, and DCOOD were obtained for surface temperatures between 228 and 273 K. The measurement of a single kinetic trace takes about 2 min and corresponds to a total dose of 0.3 ± 0.1 ML. We recorded up to ten sequential scans at every temperature to ensure that subsequent kinetic traces did not substantially deviate from one another. This was necessary as there is a slow poisoning of the surface reactivity with increased formic acid exposure.

### 2.2 Theoretical Section

DFT calculations for formic acid on Pd(111) were performed using VASP 5.3.5^14−17^ with three GGA XC functionals: PW91,^18^ PBE,^19^ and RPBE.^20^ The optimized lattice constants for the Pd crystal are *a*_0_^PW91^ = 3.948 Å, *a*_0_^PBE^ = 3.945 Å, and *a*_0_^RPBE^ = 3.986 Å. These values agree within 2% with the experimental lattice constant.^21^ For these three functionals, the plane wave energy cutoff is set to 450 eV. The Pd(111) surface is modeled as a slab of 4 (3 × 3) Pd layers arranged in ABC stacking with a vacuum layer of 20 Å. The Brillouin zone is sampled by a 8 × 8 × 1 Γ-point centered k-point mesh using Monkhorst–Pack sampling.^22^ The interaction between valence and core electrons is described by the projector augmented-wave method.^23^ Partial electronic occupations were modeled with the Methfessel–Paxton (*N* = 1) smearing scheme^24^ with a smearing width of σ = 0.1 eV. Convergence is assumed when the energy difference between two iteration steps is lower than 10^–5^ eV. Two methods are investigated to describe the van der Waals energy corrections: (1) the Tkatchenko–Scheffler^25^ method combined with PBE and (2) the DFT-D3^26^ method used with both PBE and RPBE functionals. Optimized structures were found using the conjugate gradient algorithm.

The vibrational frequencies for HCOOH were calculated using VASP 5.3.5 for each GGA functional and each dispersion correction. To calculate the Hessian matrix, we performed 4 displacements for each direction and 0.015 Å width for each nucleus. The frequencies of the deuterated isotopologues were calculated through diagonalization of the Hessian matrix, obtained for HCOOH, but replacing H atoms with D atoms as necessary for each isotopologue.

The isotope independent binding energy *D*_e_ is obtained, from DFT, by
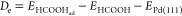
1where *E*_HCOOH_ad__, *E*_HCOOH_, and *E*_Pd(111)_ are the total energy of the formic acid adsorbed on Pd(111), of the gaseous formic acid, and of the relaxed Pd(111) surface, respectively.

## 3 Results

### 3.1 Measurement of the Formic Acid Lifetime and Determination of the Elementary Desorption Rate Constant on Pd(111)

**Figure 1:**
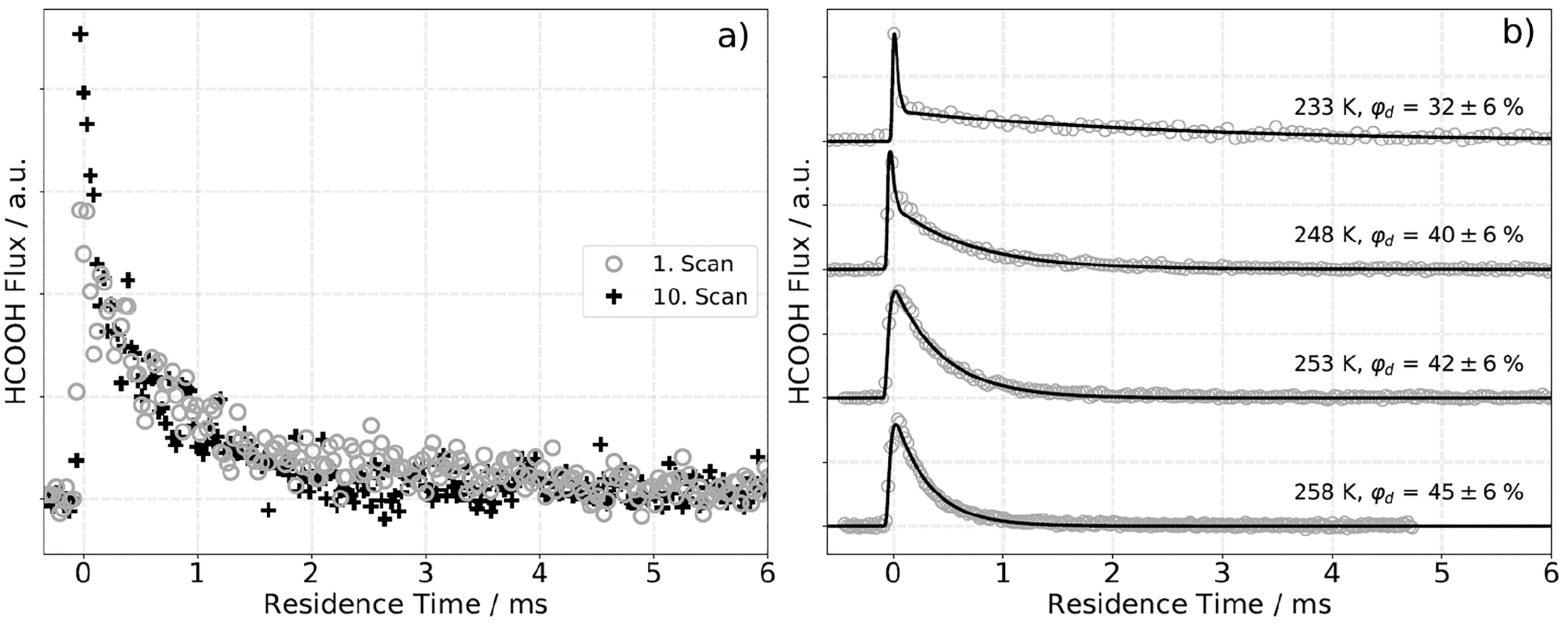
Kinetic traces of HCOOH desorption from Pd(111). (a) The gray circles show the kinetic trace of formic acid desorption after 2 min; the black crosses show the kinetic traces of formic acid desorption after 20 min. (b) Representative kinetic traces of HCOOH at four surface temperatures obtained prior to observable surface poisoning effects. φ_d_ denotes the desorption probability of HCOOH which is obtained from the integrated HCOOH desorption yield. The black solid line is a fit according to eq 2.

Typical kinetic traces measured for desorption of formic acid from Pd(111) are shown in Figure 1. They exhibit a peak at early times followed by an exponential decay. The former is assigned to directly scattered molecules (DS) and the latter to formic acid molecules that are trapped by the surface prior to desorbing (TD). As seen in Figure 1a, the shape of the kinetic trace depends on the cumulative dosing. As the exposure time increases, the relative amount of the TD component decreases, reflecting a decrease in the formic acid sticking probability. As formic acid clearly desorbs promptly from the surface, formic acid decomposition must explain this effect. Given bidentate formate’s known stability on Pd^27^ and the relatively low temperatures in our experiment, we infer that bidentate formate, produced from formic acid decomposition, effectively blocks adsorption sites. In order to avoid errors introduced by bidentate formate buildup, we acquired data over the first 6 min of formic acid exposure, where we empirically observed that surface poisoning is negligible. The surface is subsequently flashed to a high temperature prior to the next kinetic trace measurement.

The lifetime of formic acid on Pd(111) is obtained by fitting kinetic traces with the formula
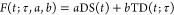
2Here, *F* is the time-dependent flux of desorbing and scattered formic acid, *a* and *b* are amplitude parameters, and τ is the lifetime of formic acid on the surface. The shape of the DS component is described by the time profile of formic acid desorption, obtained at 973 K, where the surface residence time is much shorter than the duration of the pulsed molecular beam. The time profile of the TD component is modeled as a convolution of the dosing profile with a single-exponential decay. Typical fits to the zero-coverage kinetic traces of formic acid desorption are shown in Figure 1b.

**Figure 2:**
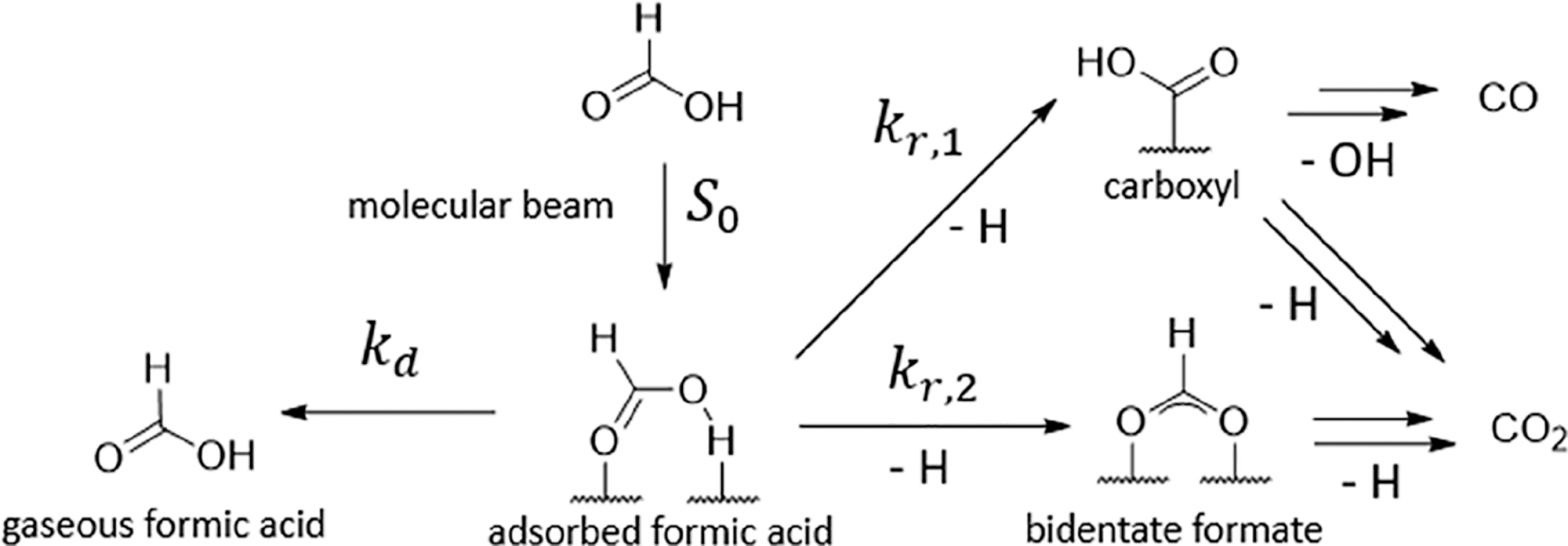
Kinetic mechanism of formic acid desorption and decomposition on Pd(111). Adsorbed formic acid can either branch into one of the two intermediates (bidentate formate and carboxyl) or desorb into the gas phase.^28^ Under our experimental conditions, we find that bidentate formate and the carboxyl intermediate are formed from formic acid irreversibly (see text). The double arrow after carboxyl and bidentate formate indicates that further intermediates may be possible for the formation of CO_2_ and CO.

Formic acid undergoes decomposition on the Pd surface, which means that the measured kinetic traces reflect the elementary rates of formic acid desorption as well as those of decomposition. The elementary reactions involved in our experiment are shown in Figure 2.

In VRK we measure the time-dependent flux (*F*_*t*_) of molecules desorbing from the surface, which in this experiment is *F*_*t*_(HCOOH_g_), given by eq 3.
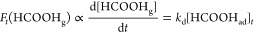
3Here, *k*_d_ is the desorption rate constant and [HCOOH_ad_]_*t*_ is the time-dependent concentration of adsorbed formic acid, described by eq 4.
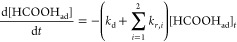
4*k*_*r,i*_ is the reaction rate constant for the *i*th decomposition pathway of formic acid.

To obtain the desorption rate constant, we have to determine the branching ratio of formic acid between desorption and decomposition. This is simplified by our observations that only CO_2_ and H_2_ are formed; no sign of CO or H_2_O was seen below ∼650 K. The zero-coverage desorption probability of formic acid is defined as given in eq 5:
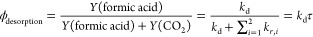
5*Y* is the yield of formic acid and CO_2_. We point out here that eq 5 assumes irreversible formation of CO_2_ forming intermediates. From theoretical calculations it can be argued that hydrogenation of the bidentate formate might be possible. However, we found that it was impossible to promote the backward reaction to formic acid, even in the presence of excess hydrogen atom coverage achieved by 10^–6^ mbar static gas of H_2_ and D_2_. Within our detection sensitivity, no backward reaction could be observed, from which we conclude that it is negligible under our experimental conditions (see Section 4 for further discussion).

The yield of any desorbing species is equal to the time, velocity, and angle integrated flux signal IS multiplied by the MPI efficiency coefficient α:
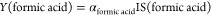
6
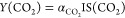
7α is different between formic acid and CO_2_. We point out that the sum of CO_2_ and formic acid yields given by
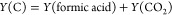
8is equal to the total number of adsorbed formic acid molecules. We make use of the C atom mass balance, given in eq 8, to determine the ratio of the proportionality constants α_CO_2__/α_formic acid_. We perform this calibration at elevated surface temperatures (403 to 523 K) where the desorption of formic acid and CO_2_ is ensured between sequential molecular beam pulses.

**Figure 3:**
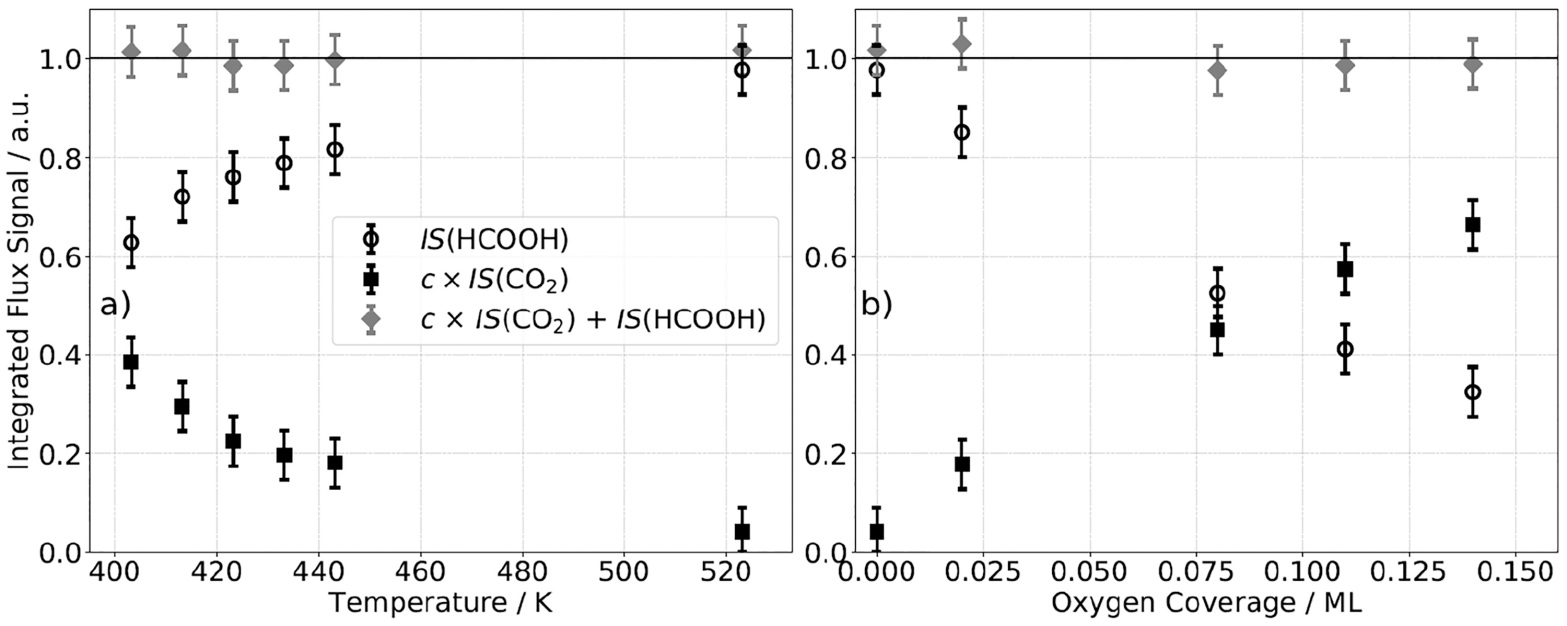
Demonstration of the mass-balance calibration for CO_2_ vs HCOOH. (a) Integrated flux signal of formic acid is correlated to the integrated flux signal of CO_2_ as a function of surface temperature. These changes allow us to determine the relative detection sensitivity between formic acid and CO_2_. We determine *c* such that the sum of the integrated flux signal of formic acid and CO_2_ is constant over the temperature range. (b) Integrated flux signal of formic acid is correlated to the integrated flux signal of CO_2_ for different surface coverages of atomic oxygen. This calibration is done at a fixed temperature of 523 K. Assuming negligible changes of the formic acid sticking probability, the parameter *c* is determined such that the sum of the integrated flux signal of formic acid and CO_2_ is constant to calibrate the relative detection sensitivity. We find that within 3% the calibration procedures yield the same result, confirming our methodology. The error bars indicate for both procedures an intrinsic error of 5%.

The calibration is done with two different methods. In the first method, we determine the sum of the integrated flux signals IS at several surface temperatures between 403 and 523 K. Combining eqs 6–8 yields
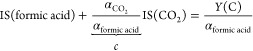
9Assuming that the sticking probability of formic acid is surface temperature independent in this temperature range, we optimize the coefficient *c* such that the sum of the flux signals IS is constant for the chosen temperature range (see Figure 3a). In the second approach, we dose the surface with small amounts of molecular oxygen which promotes formic acid decomposition. We determine the integrated flux signal of formic acid and CO_2_ as a function of surface O atom coverage. The parameter *c* for low O atom coverages is determined again by ensuring that the sum of the integrated flux signals of formic acid and CO_2_ is constant. Both approaches result in a similar value of *c* (= 1.20 ± 0.07). The constant flux of the molecular beam and a constant laser power is ensured during the calibration and subsequent desorption experiments.

The agreement between the two calibration procedures indicates that the assumption of temperature independent sticking probabilities is justified. Assuming that the sticking probability between 228 and 273 K—temperature range of desorption experiments—is unchanged from the sticking probability between 403 and 523 K, the integrated flux signal of formic acid can be directly related to the desorption probability with the following relationship:
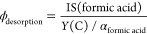
10Notice that *Y*(C)/α_formic acid_ is obtained from high-temperature experiments, while IS(formic acid) can be used from low-temperature desorption experiments.

By determination of the desorption probability of formic acid, we disentangle the contribution of the desorption rate to the measured formic acid lifetime (see eq 5).

**Figure 4:**
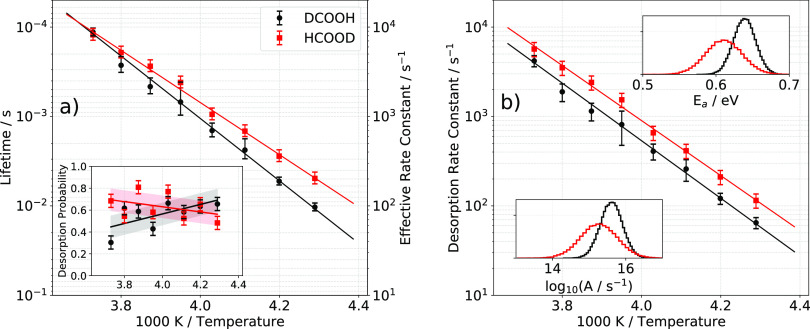
(a) Comparison of the lifetime of DCOOH and HCOOD on Pd(111). The lifetime is determined by desorption and decomposition. The inverse of the lifetime is equal to the effective first-order rate constant. The solid lines show Arrhenius fits to the effective rate constants of HCOOD (*A*^eff^ = 6.90 × 10^14^ s^–1^, *E*_a_^eff^ = 0.58 eV) and DCOOH (*A*^eff^ = 8.94 × 10^16^ s^–1^, *E*_a_^eff^ = 0.69 eV). The inset shows the desorption probabilities for DCOOH and HCOOD. Note that the inset has the same *x*-axis. The solid lines show a linear fit to reduce the scatter in the experimental desorption probabilities. The shaded areas show the 1σ uncertainty of the linear fit and is taken into account for the error estimation. (b) Desorption rate constant of DCOOH and HCOOD from Pd(111) after correction. The Arrhenius parameters are similar for DCOOH and HCOOD reflecting a similar formic acid binding energy and adsorbate entropy. The inset in the lower left shows the parameter distribution for the decadic logarithm of the pre-exponential factor and the inset in the upper right of the activation energy.

The measured lifetimes and desorption probabilities for DCOOH and HCOOD resulting from this analysis are shown in Figure 4a; note that the desorption probabilities differ for the two isotopologues. Dividing the desorption probabilities by the corresponding lifetime yields the elementary desorption rate constants shown in Figure 4b. The Arrhenius rate parameters derived from a fit to the isotopologue specific desorption rate constants are similar.

The Arrhenius parameters for the elementary desorption rate constant are summarized for all four isotopologues in Table 1.

**Table 1:** Arrhenius Parameters for the Elementary Desorption Rate Constant for HCOOH, HCOOD, DCOOH, and DCOOD from Pd(111) in the Temperature Range from 228 to 273 K

	HCOOH	HCOOD	DCOOH	DCOOD
*A*/s^–1^	10^15.4±0.3^	10^15.3±0.3^	10^15.6±0.3^	10^14.9±0.3^
*E*_a_/eV	0.62 ± 0.01	0.61 ± 0.01	0.64 ± 0.01	0.59 ± 0.02

### 3.2 Determination of the Thermal Sticking Probability of Formic Acid from the Principle of Detailed Balance

**Figure 5:**
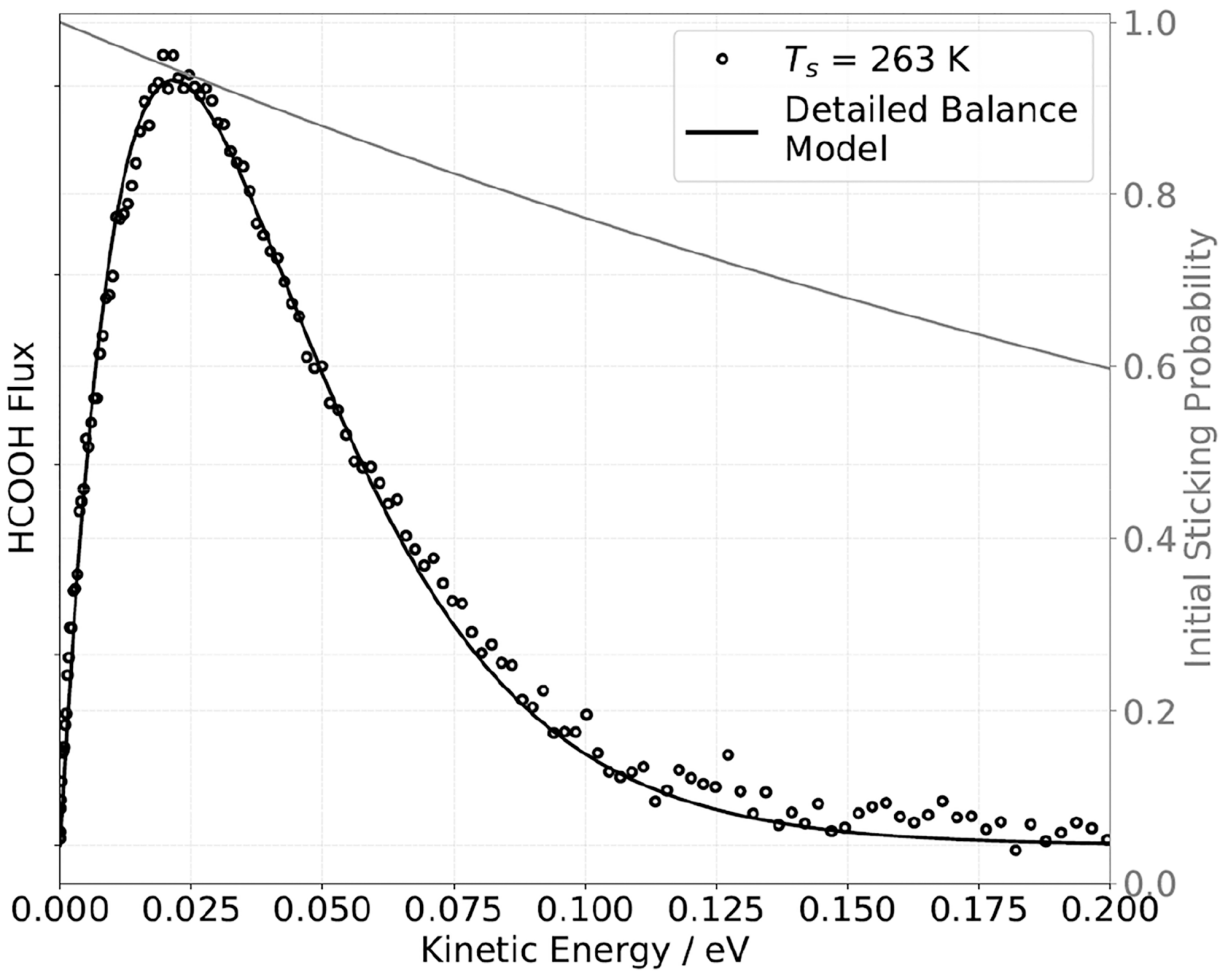
A typical flux distribution of desorbed formic acid (○) along the surface normal (θ = 0°) at 263 K. The black solid line shows the modeled kinetic energy distribution using detailed balance (eq 11) and the initial sticking probability (gray solid line, eq 12).

The analysis of these elementary rate constants within the framework of the detailed balance rate model (DBRM) is presented in Section 3.3. A crucial component of that analysis is the thermal sticking probability of formic acid at Pd(111), which we may obtain from measured speed distributions of the desorbing formic acid and the principle of detailed balance. Following the principle of detailed balance, the measured flux of desorbing molecules *F*_des_(*E*) is proportional to the product of the kinetic-energy-dependent initial sticking probability *S*(*E*) and the thermal Maxwell–Boltzmann distribution *F*_MB_(*E*,*T*_surf_) as given below:
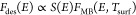
11An experimentally derived flux distribution of formic acid molecules desorbing from Pd(111) is presented in Figure 5.

The flux distribution is subthermal for all temperatures investigated, indicating that the adsorption process has no barrier. Similar to previous work,^29,30^ we used eq 12 to model the initial sticking probability of formic acid.
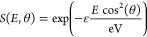
12ε is a fitting parameter, which is obtained by a global optimization routine over flux distributions derived at temperatures between 233 and 263 K. Its optimized value is ε = 2.58 ± 0.13. In this analysis, we also assume that the initial sticking probability is unity at zero kinetic energy, which was shown before a reasonable assumption for sticking without a barrier.^29,31^ Within the precision of our experiments, the initial sticking probability is indistinguishable for the different isotopologues studied here. On the basis of the extracted sticking probability along the surface normal and assuming normal kinetic energy scaling *E*_⊥_ = *E* cos^2^(θ), we use our initial sticking probability function obtained at the normal incidence *S*(*E*_⊥_,0°) to derive the angle averaged thermal sticking probability ⟨*S*⟩(*T*)^32,33^ using eq 13.
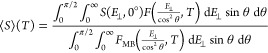
13Here, θ is the angle with respect to the surface normal. We assume cylindrical symmetry of the desorption angular distribution. The thermal sticking probability of formic acid on Pd(111) as a function of temperature is shown in Figure S3.

### 3.3 Results from DFT Calculations

**Figure 6:**
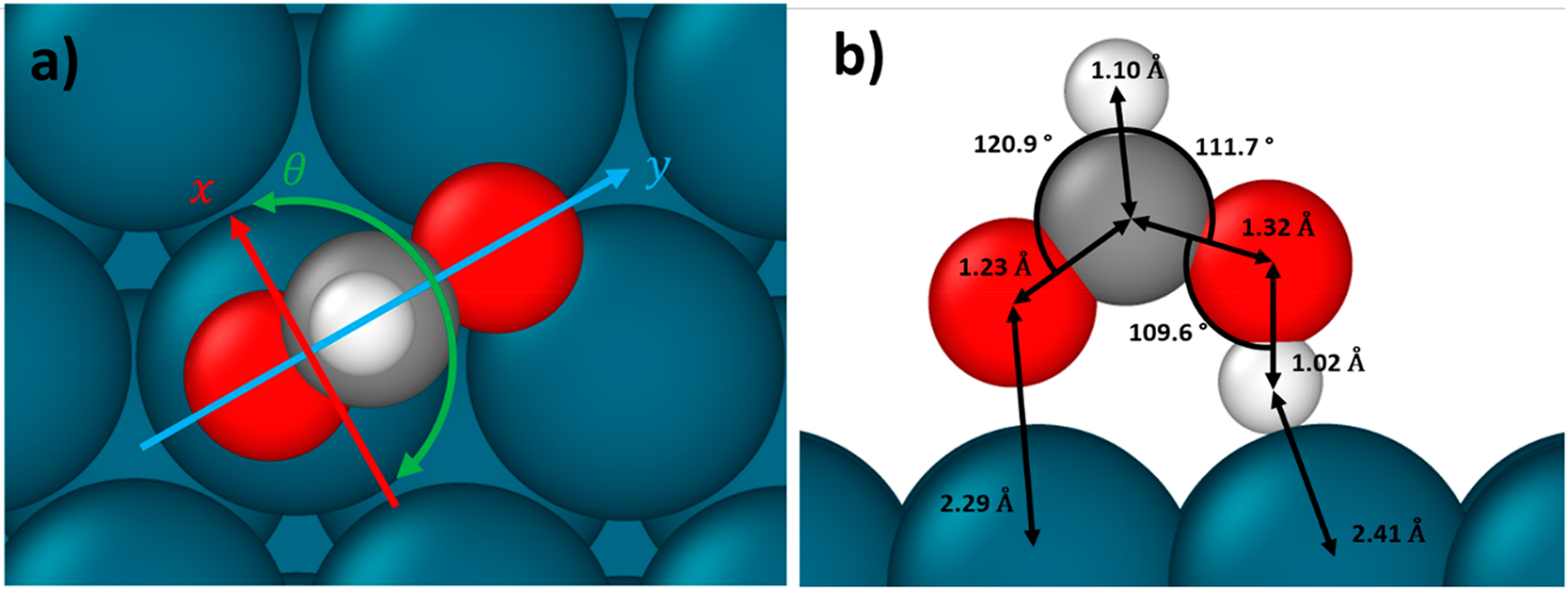
(a) Top view of the predicted minimum structure of formic acid bond on Pd(111) obtained with the PBE-TS functional. The arrows indicate the hindered translation (*x* and *y*) and hindered rotation (θ) axis of the molecule. (b) Side view with bond lengths and angles.

The most stable structure of formic acid according to the PBE-TS functional is shown in Figure 6 and is similar for all functionals used here.

Using statistical rate modeling, we want to determine the isotope-independent—classical—binding energy of formic acid on Pd(111). Therefore, we need to characterize the density of states of the adsorbate. The degrees of freedom that correlate approximately to the translational and rotational motion of the molecule in the gas phase are particularly important. The rigid rotation and translations of the molecule parallel to the surface plane are evaluated by calculating the changes in the potential energy using DFT. A periodic 1D potential adequately describes the rigid rotation of formic acid (see Figure S4). The O–H binding strength to fcc and hcp-hollow sites are very similar; hence, the rotational energy profile can be modeled as six minima within one full rotation:
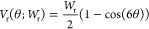
14Here, *W*_r_ is the rotational barrier. We identify the lowest barrier diffusion pathway for formic acid as the displacement along the *x*-axis. Furthermore, the minimum-energy pathway can be described with a similar 1D cosine potential employed for the rotation (see Figure S5). Other diffusion pathways, which have higher diffusion barriers, are not considered. Table 2 lists the barrier values for the rotation and the diffusion determined from a cosine function fit to the DFT energies, employing different exchange correlation functionals as well as the binding energies.

**Table 2:** Fitted Rotational Barrier *W*_r_ and Diffusion Barrier *W*_*x*_ of Adsorbed Formic Acid for Different Computational Methods^a^

	PW91	PBE	RPBE	PBE TS	PBE D3	RPBE D3
*W*_*r*_/meV	25	23	12	21	28	33
*W*_*x*_/eV	0.24	0.20	0.13	0.24	0.15	0.22
*D*_*e*_/eV	0.40	0.47	0.43	0.77	0.86	0.84

a For each method the resulting binding energy of formic acid is listed.

### 3.4 Deriving the Binding Energy and Diffusion Barrier from Detailed Balance

The Arrhenius activation energy for desorption reflects the enthalpy of adsorption at temperatures around ∼250 K, which is obviously different than the binding energy, derived from electronic structure theory. A quantitative comparison requires a model of the partition function of formic acid on Pd(111), which is implemented in our modeling of the measured thermal desorption rates.

We employ the detailed balance rate model (DBRM)^33^ to analyze the thermal desorption rate constant. Using the principle of detailed balance, the thermal desorption rate constant can be expressed as a product of thermal adsorption rate constant *k*_a_ and the equilibrium constant *K*(*T*) between gaseous and adsorbed molecules. The DBRM is formulated as follows:
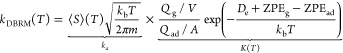
15where *Q*_g_ and *Q*_ad_ denote the partition function of the gas phase and adsorbed molecules, respectively; *V* denotes the reference volume, and *A* denotes the reference area in which the partition functions are defined; *D*_e_ denotes the classical binding energy of formic acid, and ZPE is the zero-point energy of the gas or adsorbed formic acid molecule. *D*_e_ is one of two parameters in the DBRM model that is used to fit experimentally derived desorption rate constants.

The gas phase formic acid molecule has 15 degrees of freedom; we develop the partition function as a product of individual degrees of freedom. The translational partition function is
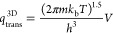
16*m* is the mass of the formic acid isotopologue, *k*_b_ is Boltzmann’s constant, and *h* is Planck’s constant. The rotational partition function for an asymmetric rotor in the high temperature limit is given by
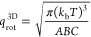
17where *A*, *B*, and *C* are the rotational constants of the particular formic acid isotopologue and are taken from ref (34). The vibrational partition function is the product of the partition function of the individual vibrations as given in eq 18.
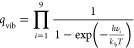
18where ν_*i*_ is the harmonic vibrational frequency of the individual modes.

Upon adsorption, the translational and rotational motion of the formic acid molecule become hindered translations and rotations. Entropy changes between adsorbates and gas phase molecules are most sensitive to these degrees of freedom. To characterize the adsorbate partition function, we conduct complementary DFT calculations (see Sections 3.3 and 4 for further discussion). The minimum-energy adsorption structure is shown in Figure 6. The carbonyl oxygen atom and the hydrogen atom of the hydroxyl group is directed toward the surface. We use the harmonic oscillator approximation to characterize the density of states resulting from the 12 degrees of freedom of the adsorbed molecule. These include nine internal molecular vibrations, the adsorbate’s hindered translation along the surface normal, and the rotations around the surface plane coordinates. We model the rotation around the surface normal (see Figure 6b) as a hindered rotor and the respective translation as a hindered translator^35^ (see below).

The partition function of the hindered rotor can be evaluated with eq 19
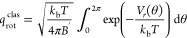
19where *B*′ is the rotational constant of the adsorbed formic acid molecule which we calculate using the DFT-optimized structure. To correct for the low-temperature limit of the classical rotational partition function, we follow the procedure introduced by Pitzer and Gwinn,^36^ whereby the classical partition function is multiplied by the ratio of the quantum harmonic oscillator *q*^qHO^ to the classical harmonic oscillator partition functions *q*^cHO^:
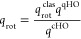
20The frequencies used in *q*_rot_^qHO^ and *q*_rot_^cHO^ emerge from the potential in eq 14 in order to maintain the self-consistency of the formula. The analytical expression for these frequencies is
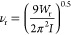
21Here, *I* is the isotopologue specific moment of inertia.

We model the translational modes (*x* and *y*) parallel to the surface with the hindered translator model.^35^ The classical formula of the hindered translator is given as
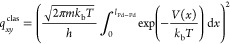
22*l*_Pd–Pd_ (= 2.77 Å) is the Pd–Pd distance on the Pd(111) surface. A simplified 1D cosine potential as given in eq 23 is used to compute the partition function. The barrier height *W*_*x*_ becomes the second optimization parameter of the DBRM model used to match to experimentally derived desorption rate constants.
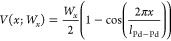
23We assume identical potentials for the *x* and *y* directions to avoid strong fit parameter correlation and again employ the Pitzer and Gwinn correction to accurately account for low-temperature behavior. We note that eq 22 describes two degrees of freedom, such that the Pitzer–Gwinn correction needs to also account for two degrees of freedom. We obtain the frequency of the hindered translator *v*_*x*_ (and *v*_*y*_) using eq 24.
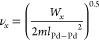
24All parameters necessary to evaluate the partition functions are given in the SI Section 1.3.

**Figure 7:**
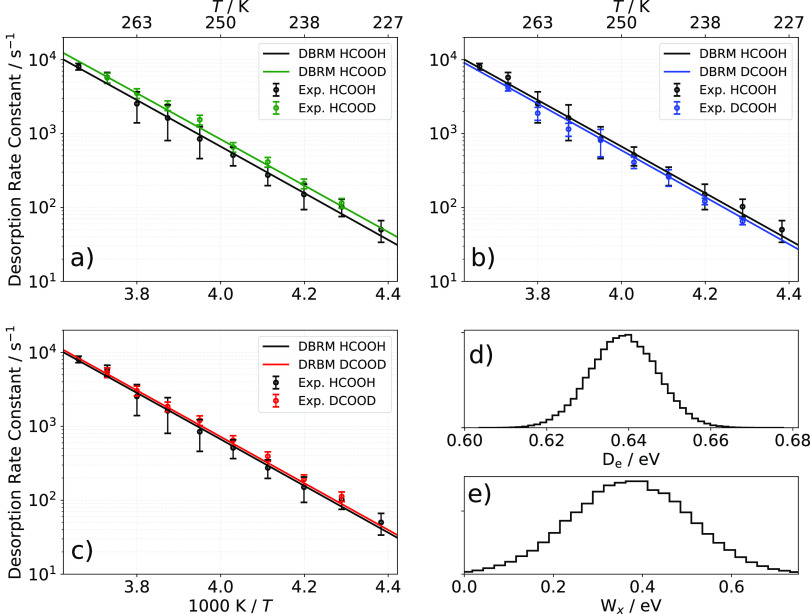
Comparison of the experimentally derived elementary desorption rate constants and the detailed balance rate model (DBRM). Experimental desorption rate constants of the different isotopologues are shown as open circles and the corresponding DBRM as a solid line: HCOOH (black, a, b, c), HCOOD (green, a), DCOOH (blue, b), and DCOOD (red, c). As a reference, we always show HCOOH to emphasize the kinetic isotope effects. The DBRM is evaluated using the harmonic frequencies of PBE-TS while the diffusion barrier *W*_*x*_ and the classical binding energy *D*_e_ are globally fitted to all isotopologues simultaneously. Although the differences between the isotopologues are very small, the DBRM is capable of predicting the right trend. In panels d and e we show the parameter distributions of the binding energy and the diffusion barrier, respectively.

Panels a–c in Figure 7 confirm the ability of our model to describe the desorption rate constants for all four isotopologues, using only two adjustable parameters: the classical diffusion barrier *W*_*x*_ = 0.37 ± 0.13 eV and the classical binding energy *D*_e_ = 0.639 ± 0.008 eV. Figure 7d,e shows the uncertainties in these two derived parameters in greater detail. Table 3 shows the isotope specific binding energies and ZPE corrections. The trends between the different isotopologues are discussed in the next section.

**Table 3:** Isotope Specific Binding Energies (*E*_0_) as Computed from the DBRM Fit to the Experimentally Derived Rate Constants^a^

	HCOOH	HCOOD	DCOOH	DCOOD
*E*_0_/eV	0.642	0.638	0.647	0.643
ZPE_g_ – ZPE_ad_/meV	3.7	–0.4	8.0	4.3

a The error bars are the 1σ confidence interval. The partition function is evaluated using DFT PBE-TS as provides the binding energy in closest agreement to the experiment. The classical binding energy of formic acid is *D*_e_ = 0.639 ± 0.008 eV, which is uniform for all isotopologues.

## 4 Discussion

We have demonstrated above how to accurately describe the formic acid desorption rate constant from Pd(111) for all isotopologues studied. We now investigate the origin of the kinetic isotope effect (KIE). Subtle difference in the desorption rate constants are observed, which can emerge from energetic or entropic effects; i.e., ZPE influences the isotope specific binding energies and changes in atomic mass influence the density of states. We find that the formic acid vibrations ν(O–H(D)), ν(C–H(D)), ν(C–O), and δ(H(D)′O′C) contribute most strongly to the ZPE-related differences in the desorption rate constants (see Table 4). Here, ν denotes a stretching mode and δ denotes a libration mode. This explains the fact that the largest isotope effect is that between HCOOD and DCOOH:
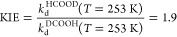
25

**Table 4:** Zero-Point Energy Difference (ΔZPE) of the Most Relevant Vibrational Modes^a^

	HCOOH	HCOOD	DCOOH	DCOOD
ΔZPE(ν(O–H(D)))/meV	40.4	31.1	40.2	31.2
ΔZPE(ν(C–H(D)))/meV	–3.9	–3.3	–0.6	–0.5
ΔZPE(ν(C–O))/meV	–10.4	–2.8	–7.4	–2.2
ΔZPE(δ(H(D)′O′C))/meV	5.2	2.0	3.8	2.3
ΔZPE_H(D)COOH(D)_ – ΔZPE_HCOOH_ (selected modes)/meV	0.0	–4.3	4.7	–0.5
ΔZPE_H(D)COOH(D)_ – ΔZPE_HCOOH_ (all modes)/meV	0.0	–4.0	4.3	0.6

a ν denotes a stretching and δ denotes a liberation mode. We do not list the vibrational modes with ΔZPE between the isotopologues of <1.3 meV. The last two rows show the ΔZPE between a particular isotopologue and HCOOH caused by the selected vibrational modes and all vibrational modes, respectively. The frequencies for the adsorbed molecule are calculated using DFT PBE-TS.

The KIE between HCOOH and DCOOD is more strongly entropic. We find that the partition function for the rotational degrees of freedom in DCOOD is ∼10% larger than that of HCOOH; this is the largest effect compared to other degrees of freedom. Hence, the DBRM predicts a slightly larger desorption rate constant for DCOOD compared to HCOOH.

Within our experimental sensitivity, no hydrogenation of formic acid decomposition intermediates—bidentate formate and carboxyl—is observed even when the hydrogen atom coverages are increased using ambient H_2_ and D_2_ gases. While on the one hand this validates our kinetic mechanism and analysis, it disagrees with the DFT predictions of Scaranto and Mavrikakis.^28^ According to that work, the formic acid decomposition reaction to bidentate formate and atomic hydrogen is endothermic, which would make the hydrogenation of this intermediate facile even at low H atom coverages. The bidentate formate/H atom product structure results in a 0.6 eV forward barrier and a 0.5 eV backward barrier. We note that in that study the products are positioned close enough that strong repulsive interactions are inevitable—more so when both oxygen atoms are positioned directly above the Pd atoms. Preliminary DFT calculations from our group regarding the bidentate formate formation indicate that letting the product structure relax appropriately increases the reverse barrier by 0.2 eV, making the reverse reaction unlikely at theses surface temperatures. We plan to investigate the reaction processes of formic acid in more detail in the future.

In Table 5, we compare the experimentally derived binding energy and diffusion barrier of formic acid to the DFT-predicted values. We note that none of the commonly used computational methods can determine the binding energy to better than 0.1 eV. In agreement with previous results from other groups that have calculated adsorption energies of formic acid, not only PBE^37^ and PW91^28^ but also RPBE without dispersion correction underestimate the binding energy, while including the dispersion correction leads to binding energies that are too large. We point out that the error in the DFT predicted binding energy is on the order of ±0.2 eV, which causes a large uncertainty in the desorption rate constant. As a result, when employing PW91 and PBE-D3 to characterize the desorption rate at ∼250 K, we find 5 orders of magnitude too high and 4 orders of magnitude too low desorption rates, respectively. Also, the established wisdom that RPBE underestimates and PBE overestimates binding energies finds no applicability for this system.^38^

**Table 5:** Binding Energy (*D*_e_) and Diffusion Barriers (*W*_*x*_) of Different DFT Functionals with and without Dispersion Correction and the Experimentally Derived Parameters^a^

	*D*_e_/eV	*W*_*x*_/eV
PW91	0.40	0.24
PBE	0.47	0.20
RPBE	0.43	0.13
PBE-TS	0.77	0.24
PBE-D3	0.86	0.22
RPBE-D3	0.84	0.15
experiment	0.639 ± 0.008	0.37 ± 0.13

a The error bars indicate the 1σ uncertainty.

Next we compare the experimentally derived diffusion barrier to several DFT predictions. This parameter is extracted from the pre-exponential factor of the desorption rate constant, which is particularly challenging to measure precisely from low temperature experiments. Consequently, we obtain a larger uncertainty for this parameter compared to the binding energy. The PW91 and PBE-TS methods yield diffusion barriers in reasonable agreement (within 1σ) with our experimentally derived value (see Table 5). Other DFT methods used here predict a diffusion barrier that is too low.

To obtain binding energies from experimental desorption rate constants, the DBRM requires DFT input from vibrational frequencies, rotational barrier, and rotational constant. This might lead to the idea that the derived binding energy is sensitive to the use of the XC functional. However, we find only a weak dependence on the binding energy when using other XC functionals (see Table S2). This is not too surprising, as relative DFT energies associated with the DBRM input parameters profit from error compensation. For the same reason, the magnitude of the calculated binding energy is more sensitive to the choice of functional than is the diffusion barrier. As a result, the relative height of the diffusion barrier can vary between ∼60% and ∼15% of the binding energy. This implies that using the fraction of the DFT calculated binding energy as a descriptor to characterize the diffusion barrier can be misleading.^39^

While DFT calculations tend to fail when it comes to absolute binding energies, the predicted trends in binding energies between different metals can still be instructive,^40^ as our below example shows. Campbell and co-workers determined adsorption enthalpies of formic acid of 0.82 ± 0.02 eV^41^ and 0.67 ± 0.02 eV^42^ for Ni(111) and Pt(111), respectively. Previous DFT calculations on (211)-facets of 10th group metals predict a binding energy trend Ni > Pt > Pd,^43^ which is correct when compared to best available experimental binding energies on (111)-facets of these metals.

## 5 Conclusions

This work demonstrates that velocity-resolved kinetics (VRK) experiments can extract accurate desorption rate constants for a reactive system such as formic acid on Pd(111). In this study, we measured the lifetimes of four isotopologues of formic acid at several surface temperatures and their corresponding desorption probabilities. We developed internal calibration procedures to account for the relative MPI detection efficiencies of formic acid and CO_2_. We modeled the adsorbate partition functions and use the detailed balance rate model (DBRM) to determine an accurate binding energy and diffusion barrier for formic acid on Pd(111). To our knowledge, this is the first time that the binding energy of formic acid is reported with such a high precision.

## Supporting Information Available

Integration of formic acid and CO_2_ flux signal; thermal initial sticking probability of formic acid; vibrational frequencies and rotational constants of HCOOH, HCOOD, DCOOH, and DCOOD from experimental gas phase studies; harmonic frequencies, moments of inertia, hindered translator and rotator modes of adsorbed HCOOH, HCOOD, DCOOH, and DCOOD (PDF)
