# Supplementary material for: Binding Energy and Diffusion Barrier of Formic Acid on Pd(111)
Source: J Phys Chem A. 2022 Dec 30;127(1):142–52. doi: 10.1021/acs.jpca.2c07414 (PMC9841570; doi:10.1021/acs.jpca.2c07414)
Supplement: Supplementary file 1 — jp2c07414_si_001.pdf [file jp2c07414_si_001.pdf]

Supporting Information

for:

The Binding Energy and Diffusion Barrier of Formic Acid on Pd(111)

Jan Fingerhut[1+], Loïc Lecroart[2+], Dmitriy Borodin[1,2*], Michael Schwarzer[1], Stefan Hörandl[1], Alexander Kandratsenka[2], Daniel J. Auerbach[2], Alec M. Wodtke[1,2,3] and Theofanis N. Kitsopoulos[1,2,4,5*]

[1]Institute for Physical Chemistry, Georg-August University of Goettingen, Goettingen 37077, Germany.

[2]Department of Dynamics at Surfaces, Max Planck Institute for Multidisciplinary Sciences, Goettingen 37077, Germany.

[3]International Center for Advanced Studies of Energy Conversion, Georg-August University of Goettingen, Goettingen 37077, Germany.

[4]Department of Chemistry, University of Crete, Heraklion 715 00, Greece.

[5]Institute of Electronic Structure and Laser – FORTH, Heraklion 70013, Greece.

[*]corresponding author: dborodi@gwdg.de, theo.kitsopoulos@mpinat.mpg.de

[+]authors contributed equally to this work

1. Supporting Information (SI)

1.1. Integration of formic acid and CO_2 flux signal

In order to obtain a quantitiy which is proportional to the yield of both species, we integrate the kinetic trace over time. In case of CO_2, we observe a two dynamical components which we account for as explained in the following. We observe a bimodal flux distribution where one component is thermal and the other hyperthermal, see Fig. S1.

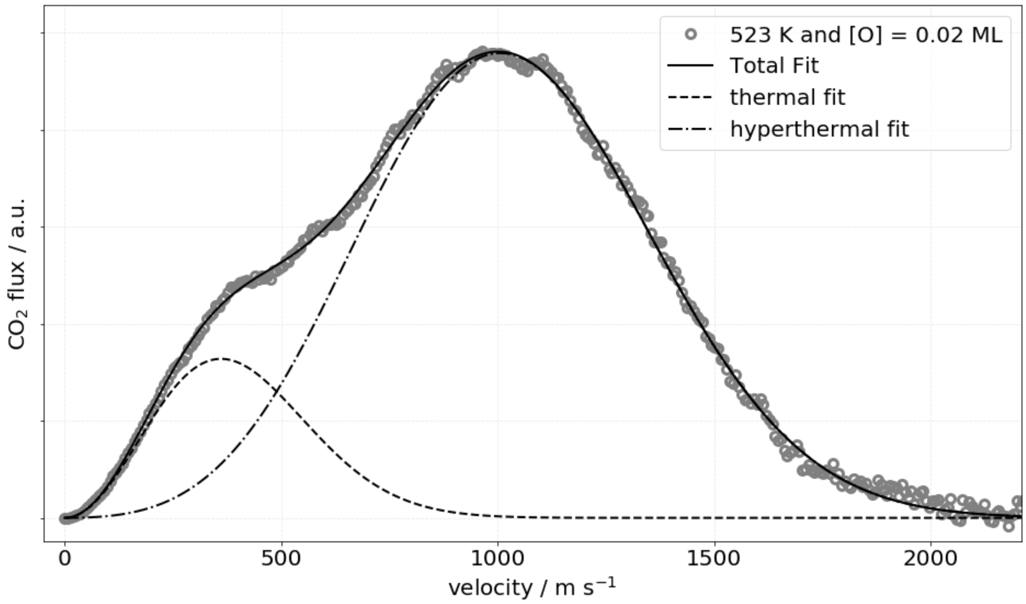

Figure S1: A typical experimental flux distribution (o) for CO_2 product following the formic acid oxidation on Pd(111) is shown. The black solid line is the total fit using Eq. S1, and the black dashed (----) and black dashed-dotted (-·-·-·) lines correspond to the thermal and hyperthermal components, respectively.

The flux distributions are extracted from the density-to-flux corrected sum image of the delay scan by integrating over a wide range of speeds. The branching between thermal and hyperthermal component is determined by fitting the experimental flux distribution with the following formula:

$$f(v; a, b, T, \beta) = a\, v^2 \exp\left(-\frac{m_{\mathrm{CO_2}} v^2}{2k_b T}\right) + b v^2 \exp\left(-\frac{m_{\mathrm{CO_2}} (v - v_0)^2}{2k_b \beta}\right) \quad \textit{Eq. S1}$$

f is the flux of CO_2, a and b are amplitude parameters, T is the effective temperature of the slow component, $m_{\mathrm{CO_2}}$ is the mass of CO_2 and β and v_0 are parameters to the hyperthermal component. Subsequently, the thermal and hyperthermal component can be integrated separately.

The angular distribution is obtained as described previously.[1] The hyperthermal component shows a sharp angular distribution ($\sim \cos^6 \theta$) while we assume the thermal component to have a $\cos(\theta)$ angular distribution. We determine the angular enrichment factor D for both components using Eq. S2.

$$D = \frac{\int_0^{\frac{5\pi}{180}} \cos^n(\theta)\sin(\theta)\,d\theta}{\int_0^{\frac{\pi}{2}} \cos^n(\theta)\sin(\theta)\,d\theta} \qquad \textit{Eq. S2}$$

The integrated desorption flux of hyperthermal CO_2 IS_{ht} is corrected by the angular enrichment and we obatin the sum of the velocity and time integrated desorption flux of both dynamical components by using Eq. S3,

$$IS_{\mathrm{CO_2}} = IS_{\mathrm{th}} + \frac{D_{\mathrm{th}}}{D_{\mathrm{ht}}} IS_{\mathrm{ht}} \qquad \textit{Eq. S3}$$

where IS_{th} and IS_{ht} are the velocity and time integrated desorption flux of the thermal and hyperthermal CO_2 obtained from the fitted flux distribution. D_{th} and D_{ht} are the angular enrichment factors of the thermal and hypterthermal CO_2, respectively.

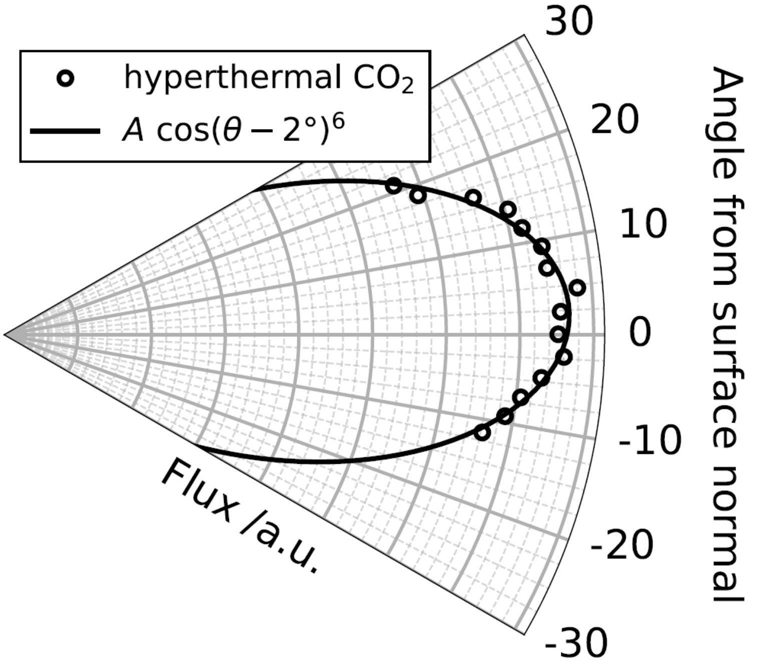

Figure S2: Typical angular distribution of hyperthermal CO_2 product following the formic acid oxidation on Pd(111) is shown. The black circles are the experimental data and the solid line corresponds to a simulated $cos^6\ \theta$ angular distribution.

1.2.Thermal initial sticking probability of formic acid on Pd(111)

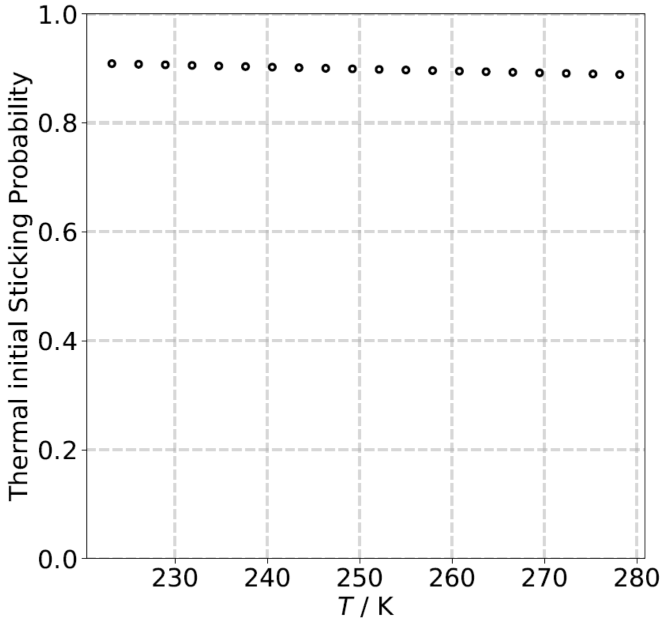

Figure S3: The thermal initial sticking probability as a function of surface temperature derived from Eq. 13.

1.3. Harmonic frequencies, rotational constants and energy profiles for translational and rotational degrees of freedom

Table S1: Listed are the DFT PBE-TS calculated frequencies, rotational constants and masses of the isotopologues HCOOH, HCOOD, DCOOH and DCOOD for the gas phase[2, 3] and the adsorbed state. We model the x and y translational modes with the hindered translator model and the R_z rotational mode with the hindered rotator model and give the corresponding frequencies $v_{x,y}$ and v_r, respectively.

	HCOOH	HCOOD	DCOOH	DCOOD
ν_g(O-H(D)) / cm^{-1}	3569	2632	3570	2632
ν_g(C-H(D)) / cm^{-1}	2942	2948	2220	2232
ν_g(C=O)) / cm^{-1}	1777	1772	1756	1742
δ_g(H(D)CO) / cm^{-1}	1381	1360	970	945
δ_g(H(D)’O’C) / cm^{-1}	1223	990	1220	1040
ν_g(C-O)) / cm^{-1}	1104	1178	1143	1171
δ_g(OCO’)) / cm^{-1}	625	541	629	556
δ_g(CH(D))) / cm^{-1}	1033	1038	867	873
δ_g(OH(D))) / cm^{-1}	642	512	625	489
$\tilde{A}$ / cm^{-1}	2.52	2.16	1.89	1.67
$\tilde{B}$ / cm^{-1}	0.37	0.36	0.37	0.36
$\tilde{C}$ / cm^{-1}	0.33	0.31	0.31	0.30
m / amu	46	47	47	48
ν_{ad}(O-H(D)) / cm^{-1}	2879	2101	2883	2099
ν_{ad}(C-H(D)) / cm^{-1}	3007	3005	2221	2223
ν_{ad}(C=O)) / cm^{-1}	1641	1635	1626	1622
δ_{ad}(H(D)CO) / cm^{-1}	1342	1331	959	1001
δ_{ad}(H(D)’O’C) / cm^{-1}	1134	956	1156	927
ν_{ad}(C-O)) / cm^{-1}	1282	1225	1269	1208
δ_{ad}(OCO’)) / cm^{-1}	640	578	634	509
δ_{ad}(CH(D))) / cm^{-1}	981	978	826	826
δ_{ad}(OH(D))) / cm^{-1}	667	525	660	574

v_z / cm^{-1}	115	113	114	112
v_x(DFT) / cm^{-1}	29	29	29	28
v_y(DFT) / cm^{-1}	49	48	49	48
v_{R_x}(DFT) / cm^{-1}	180	177	176	173
v_{R_y}(DFT) / cm^{-1}	179	169	161	154
v_{R_z}(DFT) / cm^{-1}	51	50	48	48
$\widetilde{B}'$ / cm^{-1}	0.24	0.23	0.24	0.23
$v_{x,y}$ / cm^{-1}	75	75	75	74
v_r / cm^{-1}	38	37	38	37

Fig. S4 shows the potential energy surfaces for the rigid rotation of adsorbed formic acid calculated using DFT methods (see also Fig. 6b).

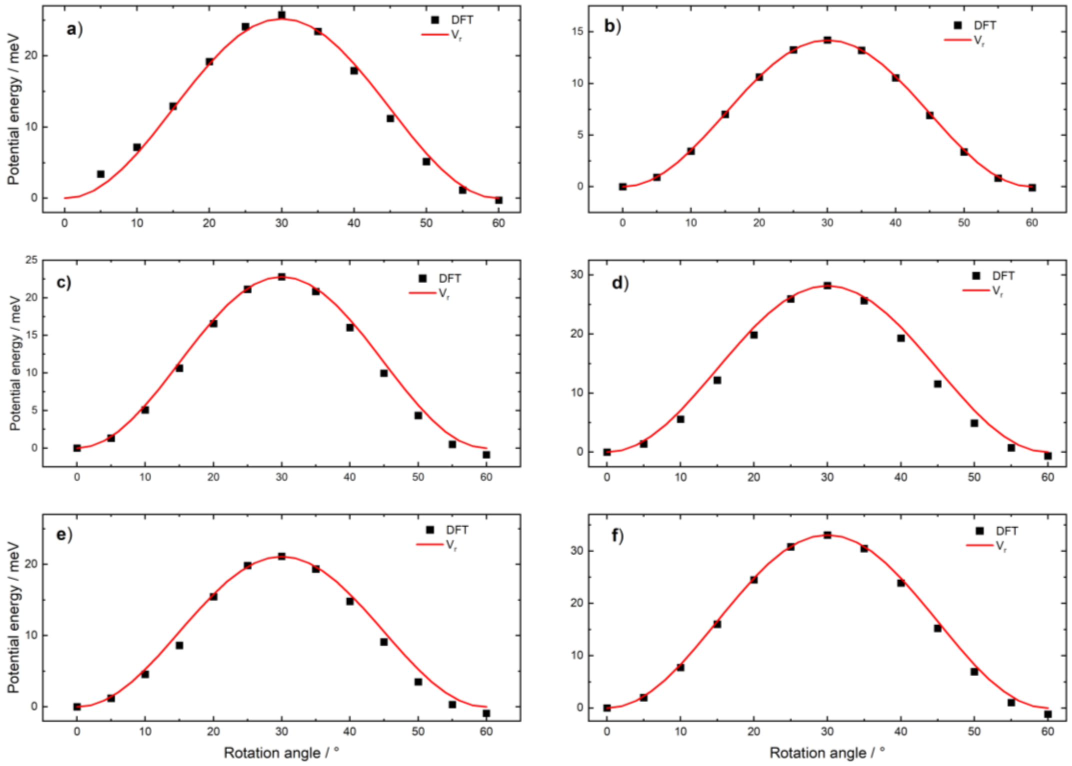

Figure S4: Calculated potential energies for the rigid rotation of adsorbed formic acid on Pd(111) as a function of the angle θ, see also Fig. 6. Black squares represent values from DFT and red solid line, the V_r potential introduced in Eq. 14, with fitted parameters. Panels a), b), c), d), e) and f) presents values for PW91, RPBE, PBE, RPBE-D3, PBE-TS and PBE-D3 methods, respectively.

Fig. S5 shows the calculated rigid translation potential energy surfaces of adsorbed formic acid about the *x* axis using DFT methods (see also Fig. 6b).

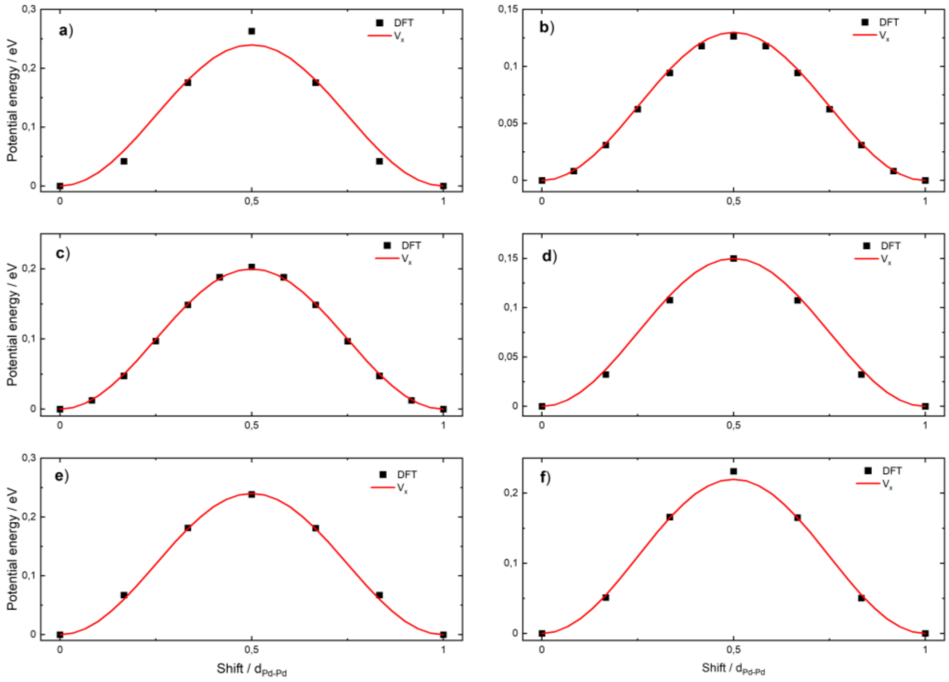

Figure S5: Calculated rigid translation potential energies of adsorbed formic acid on Pd(111)-surface along the x axis, see also Fig. 6b. Black squares represent values from DFT and red solid line, the potential introduced in Eq. 23, with fitted parameters. Panels a), b), c), d), e) and f) presents values for PW91, RPBE, PBE RPBE-D3, PBE-TS and PBE-D3 methods, respectively.

Table S2: Listed are the calculated binding energies (D_e^{DFT}) and diffusion barriers (W_x^{DFT}) using different DFT XC functionals with and without dispersion correction. Also listed are the experimentally derived parameters (D_e^{exp} and W_x^{exp}) as shown in sec. 3.4. using the different XC functionals for the input parameters. The error bars indicate the 1σ uncertainty.

	PW91	PBE	RPBE	PBE-TS	PBE-D3	RPBE-D3
D_e^{DFT} / eV	0.40	0.47	0.43	0.77	0.86	0.84
W_x^{DFT} / eV	0.24	0.20	0.13	0.24	0.22	0.15
$\mathrm{D}_e^{\mathrm{exp}}$ / eV	0.636 $\pm$ 0.007	0.640 $\pm$ 0.009	0.637 $\pm$ 0.010	0.639 $\pm$ 0.008	0.638 $\pm$ 0.007	0.641 $\pm$ 0.010
W_x^{exp} / eV	0.46 $\pm$ 0.14	0.37 $\pm$ 0.14	0.47 $\pm$ 0.16	0.37 $\pm$ 0.13	0.33 $\pm$ 0.10	0.44 $\pm$ 0.18

1. References

1. Fingerhut, J.; Borodin, D.; Schwarzer, M.; Skoulatakis, G.; Auerbach, D. J.; Wodtke, A. M.; Kitsopoulos, T. N., The Barrier for Co2 Functionalization to Formate on Hydrogenated Pt. *J. Phys. Chem. A* **2021**, *125*, 7396-7405.

2. Leach, S.; Schwell, M.; Talbi, D.; Berthier, G.; Hottmann, K.; Jochims, H. W.; Baumgartel, H., He I Photoelectron Spectroscopy of Four Isotopologues of Formic Acid: Hcooh, Hcood, Dcooh and Dcood. *Chem. Phys.* **2003**, *286*, 15-43.

3. Williams, V. Z., Infra-Red Spectra of Monomeric Formic Acid and Its Deuterated Forms .1. High Frequency Region. *J. Chem. Phys.* **1947**, *15*, 232-242.
